# Deep learning-based approach for identification of diseases of maize crop

**DOI:** 10.1038/s41598-022-10140-z

**Published:** 2022-04-15

**Authors:** Md. Ashraful Haque, Sudeep Marwaha, Chandan Kumar Deb, Sapna Nigam, Alka Arora, Karambir Singh Hooda, P. Lakshmi Soujanya, Sumit Kumar Aggarwal, Brejesh Lall, Mukesh Kumar, Shahnawazul Islam, Mohit Panwar, Prabhat Kumar, R. C. Agrawal

**Affiliations:** 1 Division of Computer Applications, ICAR-Indian Agricultural Statistics Research Institute, New Delhi, 110012 India; 2 ICAR-National Bureau of Plant Genetic Resources, New Delhi, 110012 India; 3 ICAR-Indian Institute of Maize Research, Ludhiana, 141004 India; 4 Indian Institute of Technology Delhi, New Delhi, 110016 India; 5 National Agricultural Higher Education Project, Krishi Anusandhan Bhawan-II, New Delhi, 110012 India

**Keywords:** Plant sciences, Computer science

## Abstract

In recent years, deep learning techniques have shown impressive performance in the field of identification of diseases of crops using digital images. In this work, a deep learning approach for identification of in-field diseased images of maize crop has been proposed. The images were captured from experimental fields of ICAR-IIMR, Ludhiana, India, targeted to three important diseases viz. Maydis Leaf Blight, Turcicum Leaf Blight and Banded Leaf and Sheath Blight in a non-destructive manner with varied backgrounds using digital cameras and smartphones. In order to solve the problem of class imbalance, artificial images were generated by rotation enhancement and brightness enhancement methods. In this study, three different architectures based on the framework of ‘Inception-v3’ network were trained with the collected diseased images of maize using baseline training approach. The best-performed model achieved an overall classification accuracy of 95.99% with average recall of 95.96% on the separate test dataset. Furthermore, we compared the performance of the best-performing model with some pre-trained state-of-the-art models and presented the comparative results in this manuscript. The results reported that best-performing model performed quite better than the pre-trained models. This demonstrates the applicability of baseline training approach of the proposed model for better feature extraction and learning. Overall performance analysis suggested that the best-performed model is efficient in recognizing diseases of maize from in-field images even with varied backgrounds.

## Introduction

After rice and wheat, maize (*Zea mays L*.) (Poaceae) is the most essential cereal crop grown in diverse environmental conditions across the globe^1^. In 2020, the global yield of maize was around 1.15 billion MT with a productivity of 5.82 t/ha^2^. Among the 170 maize growing countries, India stands in the fourth position in terms of maize acreage while seventh in production terms, contributing nearly 4.6% and 2.4% of the global acreage and production respectively^3^. In India, maize is grown in an area of approx. 9.02 mha with a production of 27.71 mt and productivity of 3.1 t/ha^2^. It is widely valued for its extensive use as a staple food for human beings and quality feed-fodder for animals. In addition to that maize crop also serves as the primary raw material for many industrial products. Despite a high grain yield potential, the considerable obstacle to the higher yield of the maize crop is its vulnerability to several diseases. In maize, around 65 diseases have been reported from different regions in India^4^. With respect to production loss due to the diseases, 11 out of 65 diseases are of national importance in the Indian scenario^5^. Among these Maydis Leaf Blight (MLB)/ Southern Corn Leaf Blight (SCLB), Turcicum Leaf Blight (TLB)/ Northern Corn Leaf Blight (NCLB) and Banded Leaf and Sheath Blight (BLSB) diseases are prevalent in most of the maize growing regions of India. The MLB disease is mostly favoured by the warm and humid climatic regions of India which can cause the yield loss of around 40–70% of the total production^6–8^. The TLB disease has the capability of damaging 25–90% of total crop yield in severe conditions^8^. The BLSB disease generally causes 0–60% production loss in maize in natural conditions^4^. However, the extent of yield loss may reach 100% under severely affected conditions^9^. In order to manage a specific disease of a crop, it’s very important to identify the factor correctly and then apply management tactics accordingly. Therefore, correctly identifying the disease symptoms of the crops in in-field conditions is an important step for managing the disease spread. Traditionally, domain experts/plant pathologists/farmers used to diagnose any diseases by manually visualizing the symptoms of the disease in the crops with the naked eye. However, this approach isn’t feasible to a larger extent due to the constraints like time, cost, physical accessibility and resource availability. Many times, unavailability of domain experts, may hamper the accurate treatment of the diseases in the early stage. Therefore, a precise, quick and cost-effective approach for the diagnosis of diseases in crops is a challenge for the scientific community^10^. In the present scenario, automation of disease detection using deep learning nearly outperforms the traditional disease detection methodology and provides nearly expert-level performance in critical times. Therefore, a digital image-based automatic disease identification approach in maize crop would be a practical and viable solution to reach the stakeholders like maize farmers and extension personnel of the country.

Early investigations using semantic approach^11^, rule-based and ontology-based approach^12,13^, content-based image retrieval^14^, domain-specific expert systems^15^, etc. were performed for identification of diseases and pests in several crops. These approaches have shown impactful outcomes for the agriculture sector across the globe. In the last few years, the deep learning concepts of artificial intelligence and computer vision have emerged as a potential solution for many aspects of agricultural problems^16^. The deep learning techniques, particularly the convolution neural networks (CNN) have established a trend of automatic disease identification approachs in crops using digital images^17^. For the last few years, deep learning techniques are being used to identify diseases of major crops such as Rice^18–20^, Wheat^21–24^, Tomato^25–28^, Apple^29–31^, Cucumber^32,33^, Cassava^34^, Pearl Millet^35^ etc. Mohanty et al.^36^ developed deep CNN models for automatically identifying the disease from leaf images using an open-source dataset named PlantVillage^37^. The PlantVillage dataset contains 54,306 digital images of 26 diseases from 14 different crop species captured in lab conditions. Barbedo^38^ used the pre-trained CNN frameworks to identify in-field images of several diseases affecting 12 different crops. Ferentinos^39^ worked on developing deep learning models for identifying 56 diseases of different crops from 87,848 images of leaves captured both in a laboratory (PlantVillage dataset) and in the field. Chen et al.^40^ used transfer learning approach for identification of different diseases of Rice and Maize. They used pre-trained VGGNet network for classifying the images of plant diseases. Nanehkaran et al.^41^ proposed a novel segmentation-based approach for classification of diseases of 3 different crops. In both works, authors had used images of plant diseases collected from agricultural fields.

In the maize crop, a few but significant works have been done for automatic identification of several diseases^42–49^. The authors^44–46^ have worked on developing deep learning models for identifying diseases of maize crop. They worked on the publicly available maize data from the PlantVillage repository. Authors^43,47^ used the image dataset from the PlantVillage repository for building disease classification models. However, they augmented images from other sources such as Global AI challenge, internet sources, etc. with the maize data of plant village repo. As the images of the Plant Village repository were captured under controlled environmental conditions and in a destructive manner, it limits the applicability of these approaches in real in-field conditions. DeChant et al.^42^ have developed a computational pipeline of CNNs for identifying Northern Corn Leaf Blight (NCLB/TLB) disease of maize crop. Here, they collected images of TLB disease of maize crop from the experimental field in non-destructive manner. Their approach reported promising results for identifying NCLB/TLB disease of maize. Whereas Haque et al.^48^ have used a deep CNN model i.e. ‘GoogleNet’ for identifying Maydis Leaf Blight disease from healthy leaves. However, a limitation of these works is that in both studies only one disease of maize has been addressed. Chen et al.^49^ proposed a lightweight network for recognition of eight maize diseases. They incorporated attention module with the DenseNet architecture to propose the novel model. They collected a total of 466 images of diseases of maize from agricultural fields of Fujian Province, China.

In this work, we investigated the state-of-the-art CNN framework ‘Inception-v3’ network for classifying the three diseases of maize crop along with healthy leaves. We applied different architectural layers on top of the ‘Inception-V3’ model and applied baseline training approach. With this approach, we achieved significant results for classification of diseases of maize crop. We also performed comparative analysis of the proposed approach with the pre-trained benchmark CNN models and comparatively better results.

Major contributions of this study are as follows: first, we have created an image database containing diseased (three diseases) and healthy images of maize crop. These images were collected from the standing crops in several experimental fields in a non-destructive manner. This image database was used in this study to train, validate and test the developed CNN models. Second, we employed a state-of-the-art CNN model ‘Inception-v3’ model with three architectural layers on the top and trained the models from scratch with our collected dataset. The models showed significant performance for classifying the images of maize crop even with varied complex backgrounds.

## Maize disease dataset and classification approach

### Dataset

In this experiment, a total of 5939 digital images of maize crop were captured in a non-destructive manner. The image dataset consists of three diseases classes and one healthy class. A summary of collected images of maize crop has been shown in Table 1. The images of maize crop were collected from experimental fields of All India Coordinated Research Project (AICRP) on Maize centres of ICAR-Indian Institute of Maize Research (ICAR-IIMR), Ludhiana, India (as described in Table S1). In this study, we mainly considered three major diseases of maize crop namely Maydis Leaf Blight (MLB), Turcicum Leaf Blight (TLB) and Banded Leaf and Sheath Blight (BLSB). Therefore, the experimental fields were chosen based on hotspot locations of MLB, TLB and BLSB diseases in three maize growing zones viz. North Hill Zone (NHZ), North West Plain Zone (NWPZ) and North East Plain Zone (NEPZ). In these hot spot locations, pathology trials for screening the maize diseases are already going on under the AICRP on Maize project of ICAR-IIMR. These trails are constituted under artificially epiphytotic conditions in the various hot spot locations across the country where the region-specific susceptible/tolerant cultivars of maize are artificially inoculated with pathogen inoculum. Details of the experimental trials, diseases-wise hot spot locations and inoculation techniques followed are provided in^50^.

**Table 1:** Summary of collected image dataset of maize crop.

Category	# of images
Healthy	600
Maydis Leaf Blight (MLB)	3493
Turcicum Leaf Blight (TLB)	670
Banded Leaf and Sheath Blight (BLSB)	1176
Total	5939

**Figure 1:**
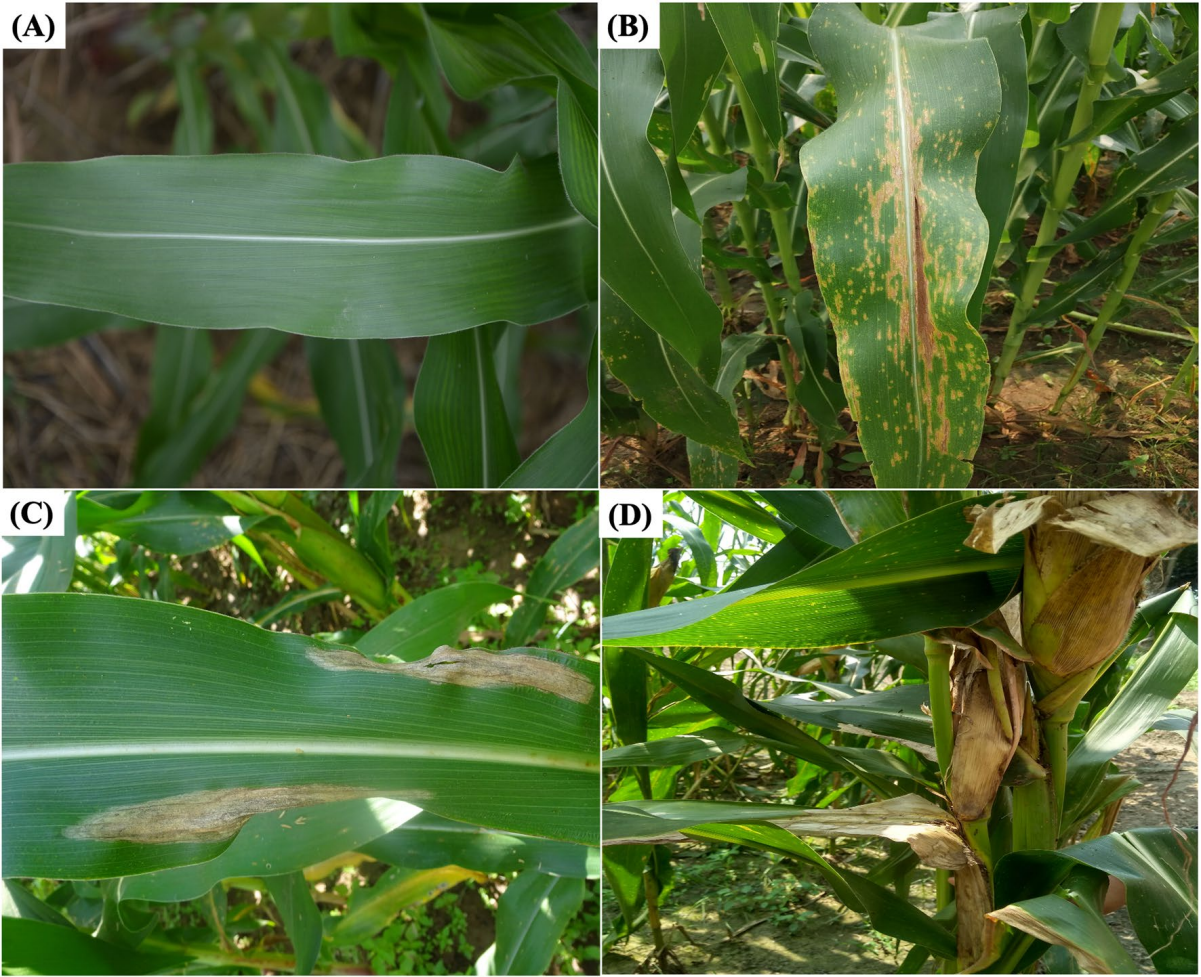
Sample images of dataset (**A**) Healthy, (**B**) Maydis Leaf Blight, (**C**) Turcicum Leaf Blight and (**D**) Banded Leaf and Sheath Blight of Maize Crop.

We collected the images of maize crop from these experimental trails during 15–60 DAS (Days after sowing) for healthy images and 25–60 DPI (days post-inoculation) for the diseased ones during both Kharif and Rabi seasons (as described in Table S1). The images of disease symptoms were associated with a mixture of susceptible and tolerant cultivars of maize crop. The variations in the symptom expressions of the diseased images were minor between the susceptible and tolerant genotypes. The images were captured manually using several image-capturing devices such as Nikon D3500 Camera having 18–55 mm Lens and CMOS Sensor with 24.2 MP; Xiaomi Redmi Y2 smartphone with 12 MP camera and ASUS Zenfone Max Pro M1 smartphone with 13 MP camera. Sample images of each diseased class have been shown in Fig. 1. While capturing the images from the maize field, the following protocols were maintained:

1. Keeping a distance of 25–40 cm between the camera lens and the plant part/leaf
2. Targeting only one affected plant part/leaf per image
3. Focusing the camera lens into the disease affected portion of the plant part/leaf
4. Capturing the top-view/front-view images of the diseased plant parts/leaves

The symptoms of the three diseases are as follows:

MLB is one of the serious fungal diseases caused by the fungus *Cochliobolus heterostrophus* (also known as *Helminthosporium maydis*). Primarily, regions with warm (20–32 °C) and humid climate favours the disease incidence. It has its effect right from the seedling stage to the harvest stage. The symptoms of this disease can be identified by the presence of small, yellowish/brown, round or oval spots on the lower or upper surface of leaves. As the disease grows, these spots enlarge, become elliptical and the center becomes straw coloured with a reddish-brown margin ^51^.

TLB disease is generally caused by the ascomycete fungus, *Setosphaeria turcica.* The symptoms of this disease start as small elliptical spots on the lower leaves which turn greenish spindle-shaped and bigger with time. The mature symptoms are characterized by 3–15 cm long cigar-shaped lesions that are gray to tan color^8^. This disease is mainly prevalent in the hilly regions of the country where the mild temperature and high humidity favours the disease development.

The BLSB is a very serious fungal disease of maize caused by *Rhizoctonia solani f.sp. sasakii*., which can wipe out the entire crop yield under severe conditions^1^. The disease generally prefers warm and humid weather conditions. The symptoms of this disease develop as straw-colored necrotic lesions alternating with dark brown on basal leaf sheaths and appear probably after 40–45 days after sowing^9^. Later these lesions enlarge and form dark brown sclerotia on diseased sheaths, husk and cobs. In severe cases, these cobs are completely damaged and dried out^9^.

### Data preparation

Pre-processing of images is an important task in the disease detection model pipeline as the images may differ in size, contain noises, have uneven illuminations, etc.^52^. As the images of maize crop were captured with different image-capturing devices (such as smartphones, digital cameras etc.), there exists variability in the images in terms of resolutions (width and height) and format. One of the fundamental aspects of deep learning networks is that the images should be homogeneous^53^. Therefore, the raw images required proper pre-processing before being applied to any of the deep learning models^52^. In this experiment, at first, all the images were resized to $256 \times 256$ pixels (resolution) by a python script using ‘*PIL*’ (Python Imaging Library) library and used for model training thereafter. All the images were saved in *.jpg* format in respective folders in storage disks. Next, images were read from the storage disks and converted to numpy arrays using the ‘*NumPy*’ library for standardization purposes. The numpy arrays provide an efficient way for reading and processing the images during model training. These numpy arrays of size $256 \times 256$ were used as input to the proposed model for feature extraction and classification.

In this experiment, it could be noted that the number of images in MLB class was far more than the other three classes. This imbalance in the number of images in the classes would have a pessimistic influence on the performance of the deep learning models. Therefore, to avoid this situation, artificial images were generated for the classes containing less number of images than MLB class. The ‘*Augmentor*’ library, available in python was used for this augmentation process. The artificial images were generated by transforming the original images in such a way that labels of the images were preserved during transformation^54^. The aim of this image augmentation process was to increase the variability as well the volume of the dataset. In this experiment, several image transformation techniques were used such as rotation (90, 270 degrees), flipping, distortion, skewing etc. A detailed summary of the augmented images has been provided in Table 2. We have also applied brightness enhancement techniques in our dataset to resolve the effect of uneven brightness in the disease symptoms and discussed in the results section.

**Table 2:** Summary of the dataset after augmentation.

Category	# of Original images	# of Augmented images	# of total Images	Augmentation details
Healthy	600	3000	3600	Rotation- 90° and 270°, Flipping Left–Right, Random Distortion and Skewed
MLB	3493	0	3493	NA
TLB	670	2680	3350	Rotation- 90°, Flipping Left–Right, Random Distortion and Skewed
BLSB	1176	2352	3528	Rotation- 90° and Flipping Left–Right
Total	5939	8032	13,971	

### Classification approach

In present scenario, deep convolutional neural networks (CNNs) are at the core in the field of computer vision and pattern recognition^55^. The CNNs have the capability to learn the distinguishable features of the images/objects automatically from pixel arrangements in the images, unlike the traditional machine learning approaches where classifiers are trained with hand-engineered features of images^34^.

**Figure 2:**
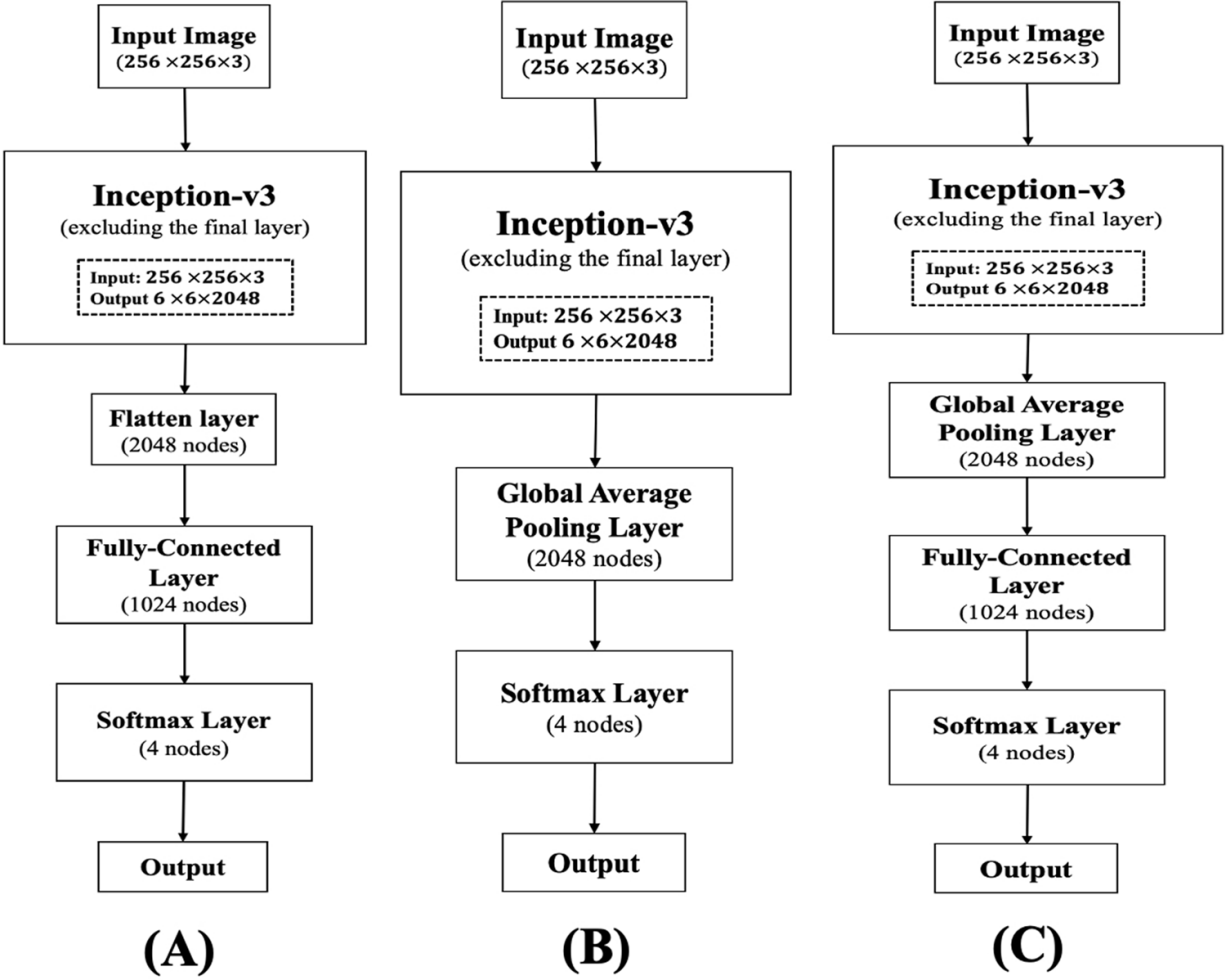
Architecture of the proposed models: (**A**) *Inception-v3_flatten-fc*, (**B**) *Inception-v3_GAP* and (**C**) *Inception-v3_GAP-fc*.

In this experiment, we used a well-known state-of-the-art CNN model ‘Inception-v3’ for classifying the in-field images of maize crop. The ‘Inception-v3’ network is 42-layers deep with concatenated convolutions and pooling layers and 2.5 times costlier than ‘GoogleNet’ in terms of computational cost. The ‘Inception-v3’ network achieved the top-5 error rate of 3.58% and the top-1 error rate of 17.2% during the evaluation with the ‘ImageNet’ dataset^55^. We employed three different architectures on top of the ‘Inception-v3’ model viz. flatten layer with fully connected layer (*Inception-V3_flatten-fc*), global average pooling layer (*Inception-v3_GAP*) and global average pooling layer with fully connected layer (*Inception-V3_GAP-fc*). The network diagrams of the proposed three models have been shown in Fig. 2. In last couple of years, past studies have shown the effectiveness of Inception network (pre-trained/ base line trained) in recognizing the diseased images of crops^26,27,34,36,38,43,48^. It has also been observed that, ‘inception’ module of Inception network integrated with other benchmark deep CNN models obtained significant results for disease identification problems^30,31,40^. Keeping in mind the image recognition performance on disease classification in the previous studies, we chose inception-v3 as the base network for this experiment.

We evaluated and compared the performances of these models on the collected in-field image dataset of maize crop. We applied the baseline training approach where the all the layers of the models were trained with our maize dataset. We didn’t use any pre-trained weights for the models, rather weights were randomly initialised during training time. The models were trained for 200 epochs with a batch size of 64 for both training and validation datasets. We have provided an ablation study in the result section for choice of the epochs and batch size for the training phase. The adam optimizer with the default value of the hyperparameters such as learning rate of 0.001, beta_1 of 0.9 and beta_2 of 0.999 was used. During the training, categorical cross entropy function was used as the loss function.

We also trained few pre-trained state-of-the-art deep learning models such as VGG-16, VGG-19, Inception-v3, ResNet-50-v2, ResNet-101-v2, ResNet-152-v2 and InceptionResNet-v2 with our maize dataset. We employed transfer learning on these pre-trained models by using pre-trained ‘ImageNet’ weights that are available with Keras. We compared the performances of the pre-trained models with the approach proposed in this manuscript to showcase the impact of using baseline approach in a state-of-art of the deep CNN model.

### Implementation

We implemented all the models using Keras^56^, a high-level API for the TensorFlow engine^57^ in the python programming language. All the experiments were conducted on the NVIDIA DGX GPU servers equipped with high-speed Tesla V100 GPUs. The details of the hardware and software configuration have been given in Table 3.

**Table 3:** Hardware and software configuration.

Name	Parameters
System	Nvidia DGX Server
Operating system	Ubuntu 18.04.3 LTS
CPU processor	Intel(R) Xeon(R) CPU E5-2698 v4 @ 2.20 GHz
Graphics processor unit (GPU)	Tesla V100-SXM2- 32 GB
RAM	528 GB
Deep learning framework	Keras with tensorflow on background
Deep learning environment	Jupyter Notebook
Programming language	Python

## Results and discussion

### Performance metrics

We divided the whole dataset into three partitions- training, validation and testing sets for conducting a fair performance evaluation. First, we divided the whole dataset into two parts with the ratio of 85:15 where 85% of the data were used for training and validation purpose and remaining 15% was kept separate for testing/evaluating the models after training. Next, we split the 85% data into two parts with different combinations for training-validation tasks. The summary of different training-validation configurations of the dataset is provided in Table 4. Here, we partitioned the original and the augmented data separately and combined thereafter to ensured that each partition must contain images from original dataset as well as the augmented ones. All the data splitting was done by a python script. After training and validation, the models were tested with the separate 15% testing dataset in all the experiments. It may be noted that the data used for testing all the models contains images from the original as well as augmented dataset. Then, we constructed confusion matrices to get disease-wise classification performance of the models. The confusion matrix gives the following variables:

- True positive (TP): # items predicted positive and it is true
- True Negative (TN): # items predicted negative and it is true
- False Positive (FP): # items predicted positive and it is false
- False Negative (FN): # items predicted negative and it is false

**Table 4:** Summary of dataset partitioning.

Data partition		Description
85	50–35	Training data: 50%; Validation data: 35%
	55–30	Training data: 55%; Validation data: 30%
	60–25	Training data: 60%; Validation data: 25%
	65:20	Training data: 65%; Validation data: 20%
	70:15	Training data: 70%; Validation data: 15%
15		Testing data: 15%

Next, the performance metrics such as precision, recall, accuracy and f1-score were also computed from the values of true positive (TP), false positive (FP), true negative (TN), and false negative (FN) for further evaluating models in this study^58^:

- $Prescion = \frac{TP}{{TP + FP}}$, measures the % of predicted as positives are actually positive
- $Recall = \frac{TP}{{TP + FN}}$, measures the % of actual positive are predicted as positive
- $Accuracy = \frac{TP + TN}{{TP + FP + TN + FN}}$, measure the overall performance of the model
- $F_{1} Score = 2*\frac{Precision*Recall}{{Precision + Recall}}$, measures the robustness of the model

### Performance analysis of proposed models

#### Comparative analysis of the models in different data configurations

**Figure 3:**
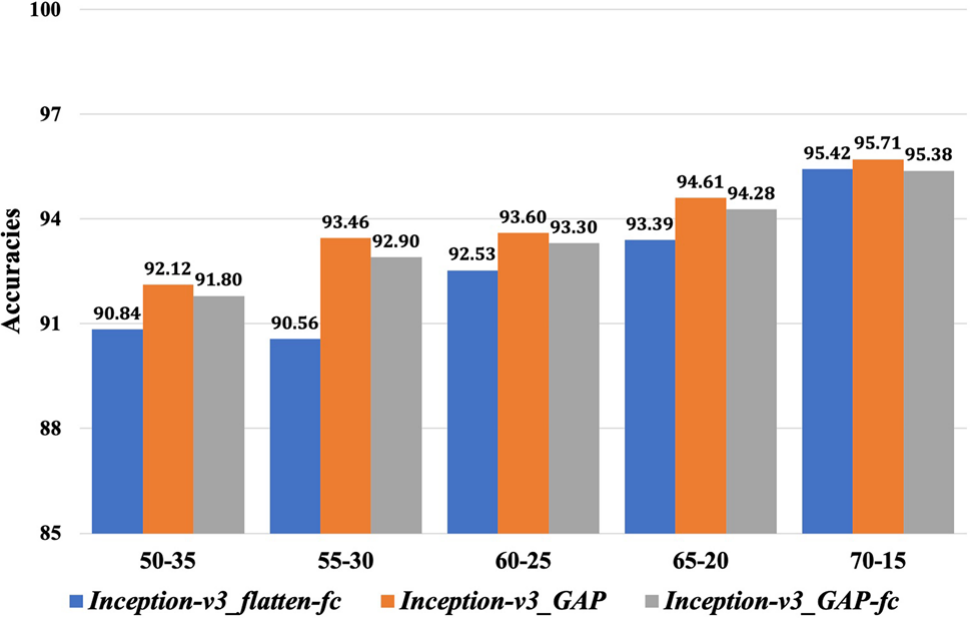
Overall testing accuracies of the proposed models on different training-validation data configurations.

The overall testing accuracies for classifying the images into correct diseased-classes for different training-validation data configurations have been provided in Fig. 3 (please see Table S2 in the supplementary file for more details). The reported accuracies ranged from 90.84% (for *Inception-v3_flatten-fc* in 50–35 data configuration) to 95.71% (for *Inception-v3_GAP* in 70–15 data configuration). It can be observed from Fig. 3 that classification accuracies of all the models showed an increasing trend as the training data were increased accordingly. The models achieved maximum accuracies when 70% of whole dataset was used for training purpose. From Fig. 3, it can be seen that *Inception-v3_GAP* model reported the higher testing accuracies consistently than the other two models (*Inception-v3_flatten-fc* and *Inception-v3_GAP_fc*) in all the data configurations. The results implied that deep learning models worked well on the images even with complex backgrounds (such as soil, straw, human body parts, other plants parts and so on) and showed reasonably better results than random guessing. These results also support the fact that deep learning models require huge number of training samples to learn and capture the inherent features from the data under study.

#### Comparative analysis of the models in 70–15 data configuration

**Figure 4:**
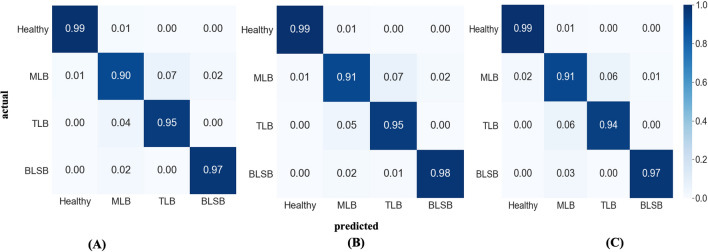
Confusion matrices of the proposed models on the 70–15 data configuration (**A**) *Inception-v3_flatten-fc* (**B**) *Inception-v3_GAP* and (**C**) *Inception-v3_GAP-fc*.

**Figure 5:**
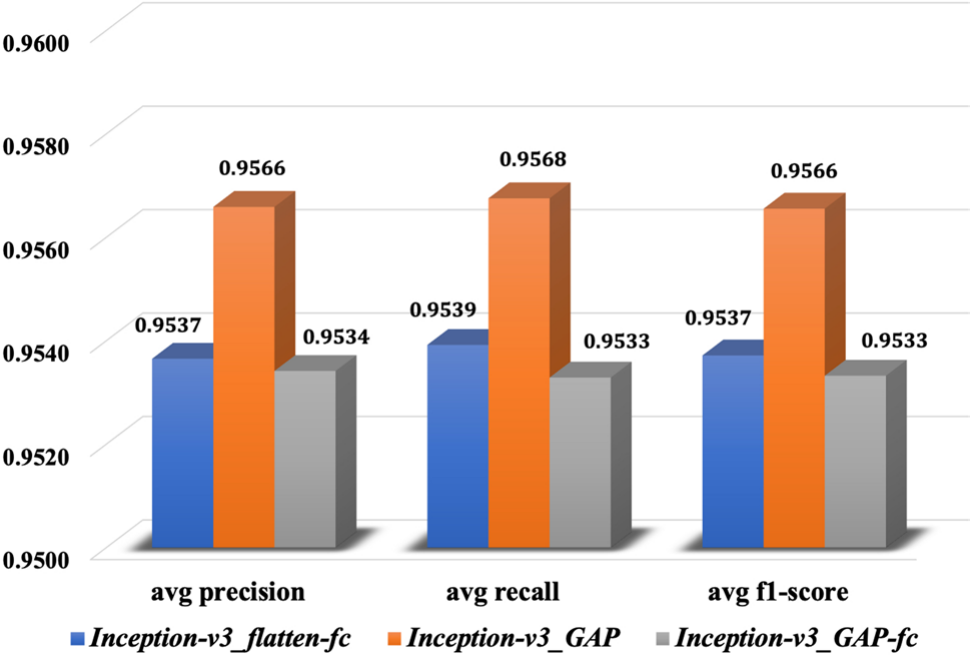
Average precision, recall and f1-score of the proposed models on 70–15 data configuration.

The confusion matrices for the models trained on the 70–15 data configurations have been portrayed in Fig. 4. The diagonal elements of the matrix represent the proportion of predictions of the trained models that matched correctly with the class levels of test data and off-diagonals represent the incorrect predictions. The confusion matrices indicate that models were good at predicting the images of Healthy and BLSB class of maize dataset, while the models weren’t quite promising for the MLB and TLB classes. After 200 epochs of training, the *Inception-v3_GAP* model achieved the overall testing accuracy of 95.71% which was approx. 0.3% higher than other two models as shown in Table 5. The testing loss of the *Inception-v3_GAP* model was 0.1861 that was lowest of all. Therefore, it is evident that *Inception-v3_GAP* showed comparatively better performance for predicting the diseases of maize crop than the other two models. The class-wise prediction accuracies of *Inception-v3_GAP* model were reported as 99% for Healthy, 91% for MLB, 95% for TLB and 98% for BLSB. Here, the reason for low accuracy in MLB class might be the similarity of initial symptoms with the TLB diseases, in turn, the model wasn’t able to distinguish the features in such images and made false predictions. We also calculated the average precision, average recall and average f1-score for the models as shown in Fig. 5. It can be noted from Fig. 5 that *Inception-v3_GAP* model achieved highest values for these metrics such as 95.66% for average precision, 95.68% for average recall and 95.66% for average f1-score. Hence, these results supports the efficiency of the *Inception-v3_GAP* for correct predictions of the targeted diseases of maize crop.

**Table 5:** Accuracy and losses of the models in the 70–15 data configuration.

Model	Accuracy	Loss
*Inception-v3_flatten-fc*	95.42	0.2419
*Inception-v3_GAP*	95.71	0.1861
*Inception-v3_GAP-fc*	95.38	0.2553

**Figure 6:**
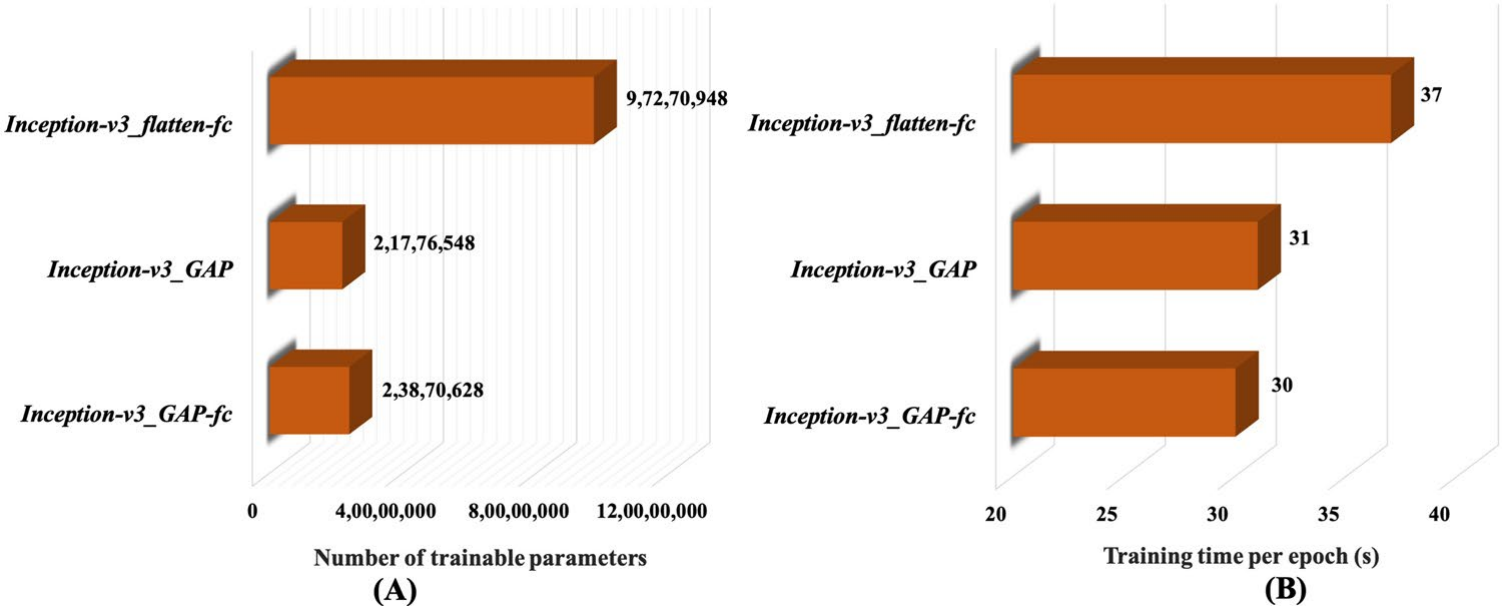
Computational behavior of the proposed models on 70–15 data configuration (**A**) Number of trainable parameters and (**B**) Training time per epoch.

Next, we analysed computational cost of these models in terms of training time per epoch and number of trainable parameters as showed in Fig. 6. It is evident from Fig. 6A that *Inception-v3_GAP* has the lowest number of trainable parameters, while *Inception-v3_flatten-fc* has the highest number of trainable parameters. And Fig. 6B indicates that both *Inception-v3_GAP* and *Inception-v3_GAP-fc* models took almost similar training time per epoch, whereas *Inception-v3_flatten-fc* took maximum time for training. This indicates that *Inception-v3_GAP* is superior than the other two models in terms the computational complexity.

#### Comparative analysis of *Inception-v3_GAP* with pre-trained models

**Figure 7:**
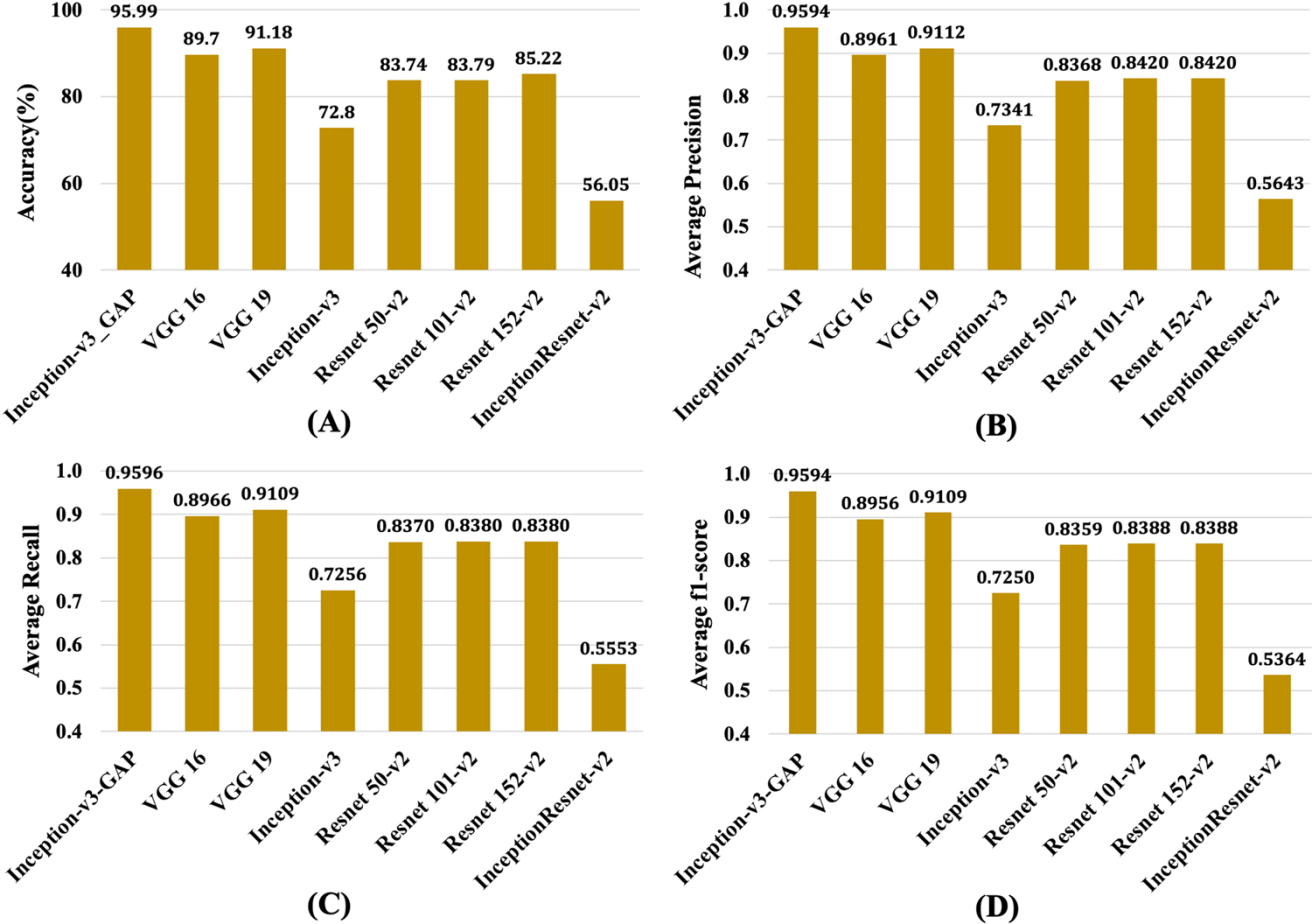
Comparative analysis of classification performance of *Inception-v3_GAP* model with pre-trained models on 70–15 data configurations: (**A**) Classification Accuracy (**B**) Average Precision (**C**) Average Recall and (**D**) Average f1-score.

We presented comparative results between the pre-trained models and *Inception-v3_GAP* model in Fig. 7 (see Table S3 in the supplementary file for more details). The pre-trained models were trained on the 70–15 data configuration and tested on the 15% testing data of the maize dataset. It is apparent from Fig. 7A that among pre-trained models, VGG 19 achieved highest accuracy of 91.18% however, Inception-v3 achieved 72.8% and InceptionResnet-v2 achieved the least (56.05%). The results show that highest performing VGG 19 is 4.53% behind the *Inception-v3_GAP* in classification accuracy. From Fig. 7B,C,D, it is clear that performance of *Inception-v3_GAP* model was way better than pretrained models in terms of average precision, recall and f1-score. The highest performing pretrained model i.e. VGG 19 reported the average precision, recall and f1-score of 91.12%, 91.09% and 91.09%, respectively, whereas the *Inception-v3_GAP* reported these metrics as 95.66%, 95.68% and 95.66%. These results indicate that it is possible to achieve better classification performances in baseline training of deep learning models than the transfer learning on pre-trained ones. It suggests that baseline learning approaches can be remarkable in extracting low-level as well as high-level features from the image dataset under study.

**Figure 8:**
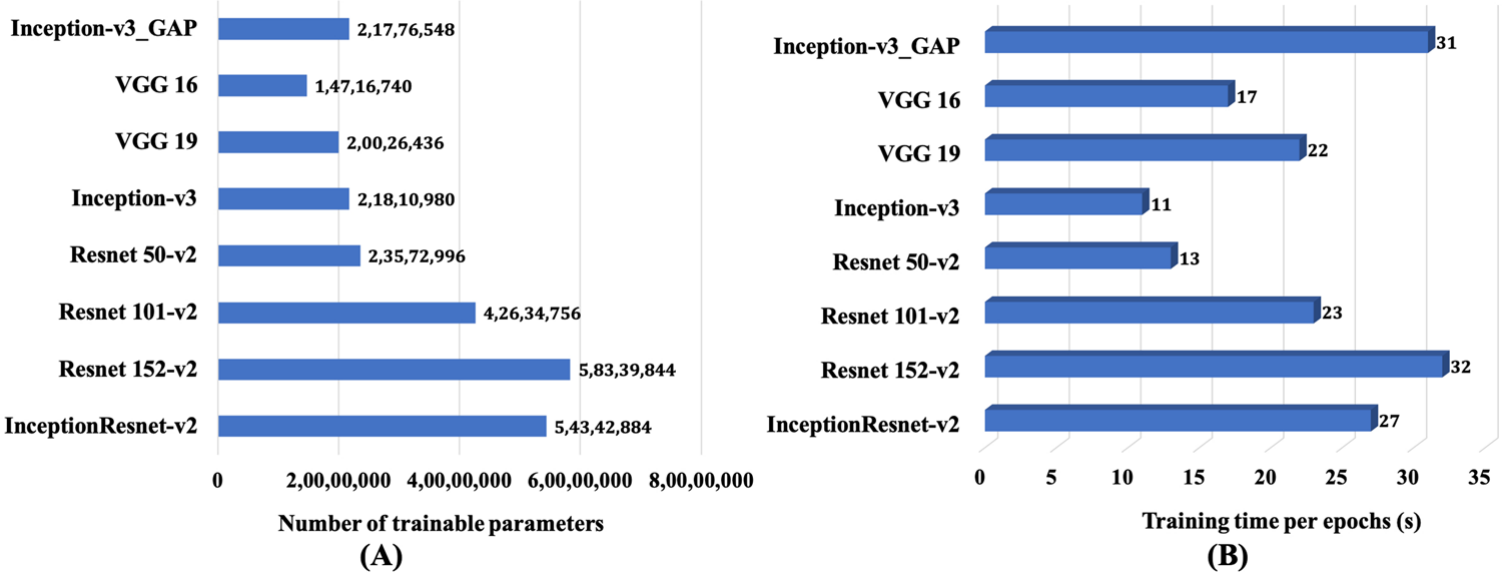
Comparison of computational behavior of *Inception-v3_GAP* model with pre-trained models on 70–15 data configuration: (**A**) Number of trainable parameters and (**B**) training time per epoch.

We also observed the computational behaviour of pre-trained models while training on the maize data. The number of trainable parameters and training time per epoch are presented in Fig. 8. It can be noted from Fig. 8A that VGG 16 has lowest number of trainable parameters and Resnet-152-v2 had highest number of trainable parameters. Whereas *Inception-v3_GAP* has approx. 48% more parameters than VGG 16 which almost similar to that of pre-trained Inception-v3. In Fig. 8B we observed an interesting fact that *Inception-v3_GAP* took 31 s per epoch, whereas Inception-v3 took only 11 s per epoch during training. Therefore, from computational point view, *Inception-v3_GAP* is slightly costlier than the pre-trained models. The main reason for this high computational cost in *Inception-v3_GAP* may be the baseline training i.e. training each and every layer of the model for feature extraction. This can be considered as a limitation of this approach. Despite this computational bottleneck, *Inception-v3_GAP* has greater advantage of higher classification accuracy in disease symptoms identification than the pretrained ones.

#### Performance analysis of *Inception-v3_GAP* model under enhanced brightness

**Figure 9:**
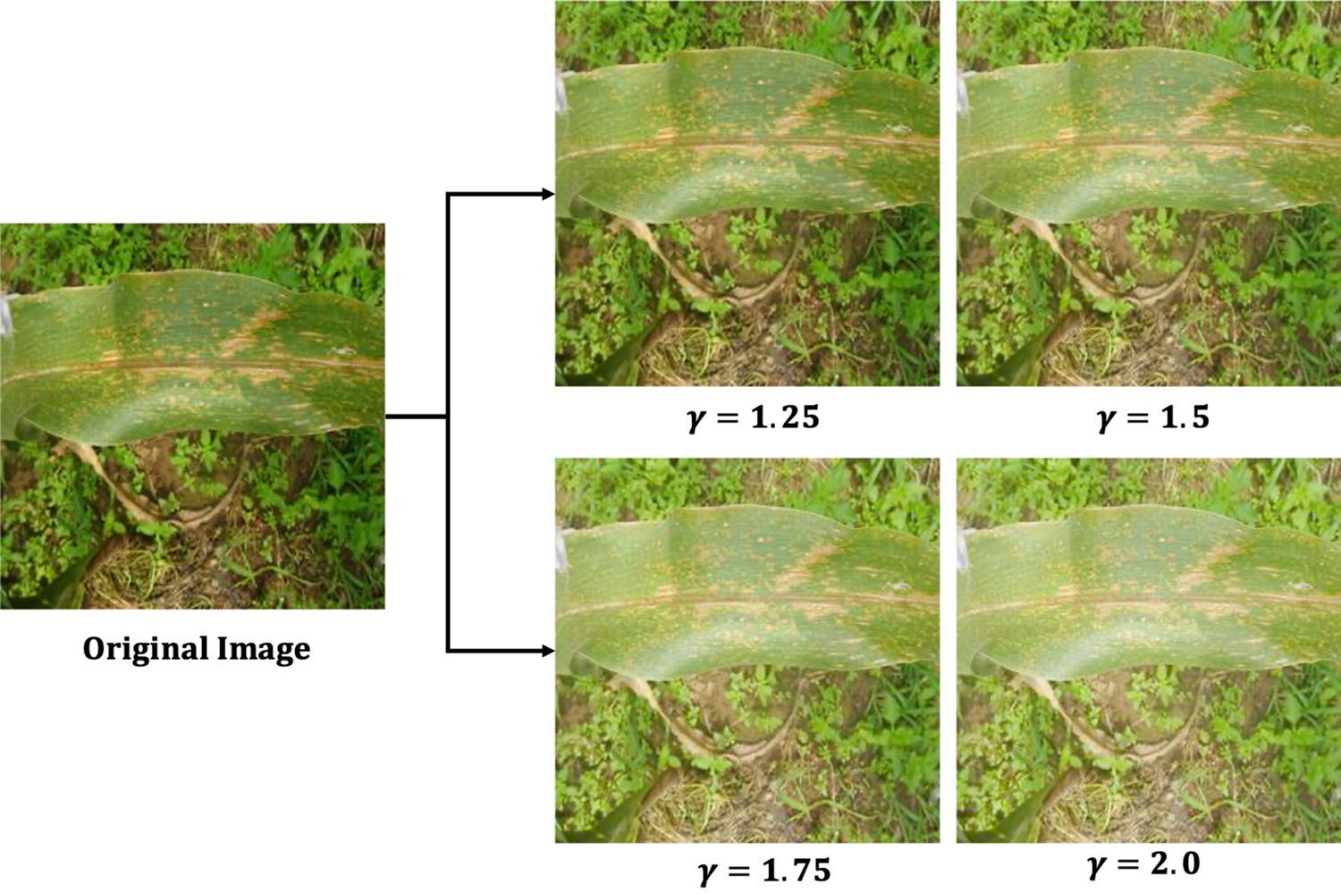
Brightness enhancement of sample images using four gamma (γ) values.

We conducted experiments on enhancing the brightness levels of the images of maize crop. As we know that in field condition, images of diseases may suffer from uneven illumination. This will affect the perception of disease symptoms within the images and ultimately affect the model’s performance while identifying the brighter images. Therefore, we applied gamma correction technique in our whole dataset to enhance the brightness levels. The gamma correction is a power-law image transformation technique^59^. We applied the gamma $\left( \gamma \right)$ operation at four levels [1.25, 1.5, 1.75 and 2.0] on the dataset and augmented with original dataset as shown in Fig. 9. Next, we trained the proposed *Inception-v3_GAP* model on this brightness enhanced dataset and presented the experimental results in Table 6. As observed in Table 6, the testing accuracy achieved by the model was 95.99% on this data with the loss of 0.1787. We can see that model’s classification performance slightly improved when data was augmented with brightness-enhanced images. This result indicates the effectiveness of the proposed model in identifying the diseased images even in the enhanced brightness condition. Hence, it can be concluded that the Inception-v3_GAP model is quite capable of capturing the features of the disease symptoms in both normal light as well as enhanced brightness condition.

**Table 6:** Performance of *Inception-v3_GAP* model on brightness enhanced data.

Testing accuracy	Loss	Precision	Recall	F1-Score
95.99	0.1787	0.9594	0.9596	0.9594

Based on the overall performance analysis, it is observed that *Inception-v3_GAP* model was better than other two models for classifying the maize diseases. This model has shown quite significant results with respect to both prediction accuracy as well as computational complexity. The GAP layer in the ‘inception-v3' model has enhanced the feature extraction and learning capability of the model. The reason behind this fact is that the global average pooling layer enforces a close relationship between the feature maps and the class levels of the problem under study. It eases out the interpretation of the class labels from the feature maps becomes feasible and lowers testing loss during the predictions. Another advantage of GAP layer is that it doesn’t add any extra parameters to the model during training, hence there is very less chance of overfitting at this layer. Therefore, it ultimately lowers the model’s trainable parameters and yet gives better prediction results. On the other hand, the *fc* layer adds a no. of trainable parameters, which increases the risk of overfitting in the models during training. Hence, the classification performance of the models with *fc* layer was lower than the GAP model. Therefore, based on the empirical analysis, it can be concluded that the proposed *Inception-v3_GAP* significantly identifies the images of diseases of maize crop.

### Comparative analysis with previous works on maize disease identification

Here, we presented a comparative analysis between our proposed approach and the approaches proposed in literatures^40,44,46,49^ for identification of diseases in maize crop in Table 7. From this comparison, it is quite evident that our proposed model is quite good at identifying the images of three diseases of maize crop even with complex backgrounds.

**Table 7:** Comparison between proposed approach and approaches available in literatures for maize crop.

References	Classes	Dataset	Data source	Models	Accuracy (%)
Chen et al.^40^	4	Own collected dataset	In-field condition	VGGNet + inception	80.38
	4	PlantVillage dataset	Lab-condition	VGGNet + inception	84.25
Marwaha et al.^46^	4	PlantVillage dataset	Lab-condition	Author-defined CNN	90.80
Sibiya et al.^44^	4	PlantVillage dataset	Lab-condition	Author-defined CNN	92.85
Chen et al.^49^	8	Own collected dataset	In-field condition	Mobile-DANet Network	95.86
Our work	4	Own collected dataset	In-field condition	Inception-v3_GAP	95.99

### Ablation studies

**Figure 10:**
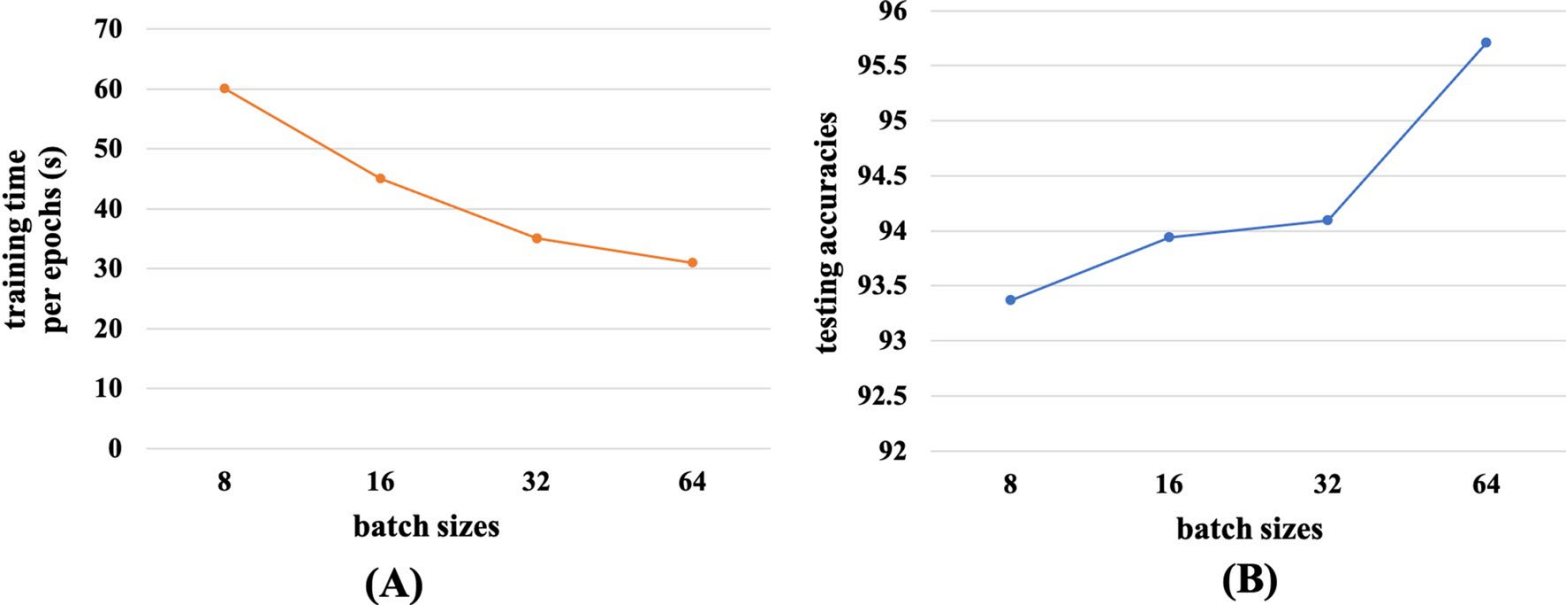
Effect of batch sizes in the model performance (**A**) batch size vs training time per epoch and (**B**) batch size vs testing accuracy of the model.

**Figure 11:**
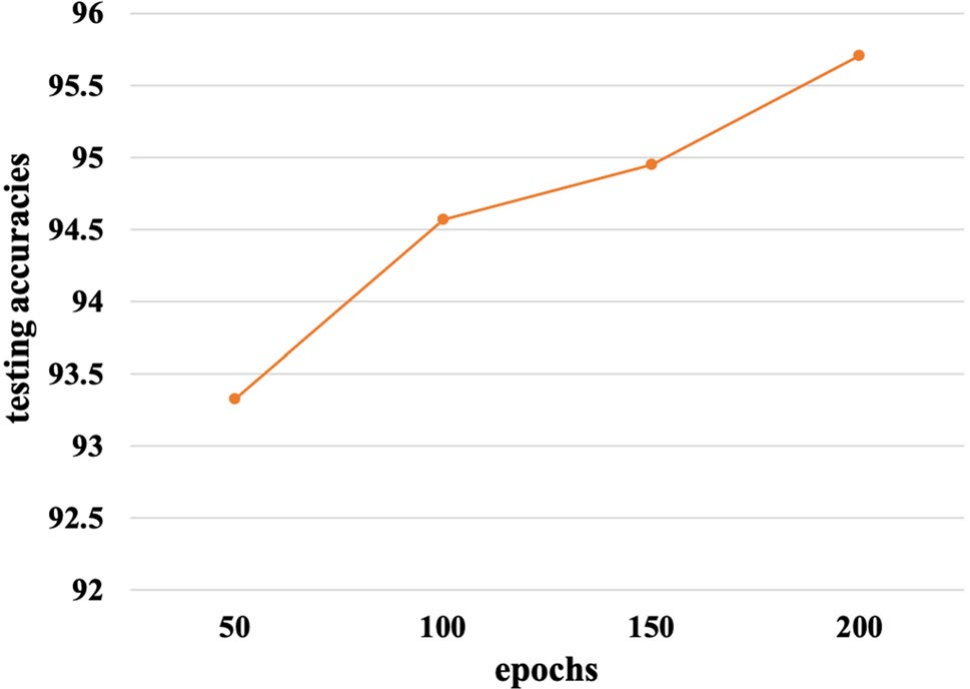
Effect of epochs in the testing accuracies.

In order to select the optimum batch size and epochs during the model training time, we conducted some experiments on different batch sizes and epochs. We presented the experimental results of  *Inception-v3_GAP* model trained with four batch sizes of 8, 16, 32 and 64 images in Fig. 10. It can be observed from Fig. 10A,B that the training time per epoch decreases, while the testing accuracy increases as the batch sizes were increased. The optimum results were obtained with 64 batch size during the model training. In Fig. 11 testing accuracies were measured at different epochs of model training. It was noticed that the testing accuracy gradually increases with epochs till it reaches 200. After 200 epochs, the model starts overfitting with the dataset, so the epochs were chosen for 200 iterations.

## Conclusions

In this study, deep convolutional neural network-based approach has been proposed to automatically identify digital images of diseases along with healthy leaves of maize crop. A total of 5939 in-field images of maize crop were collected from the experimental fields located in three maize growing zones. This dataset consists of images of three diseases such as Maydis leaf blight (MLB), Turcicum leaf blight (TLB) and Banded leaf and sheath blight (BLSB) along with healthy ones. Images were collected using different devices such as handheld camera, smartphones to incorporate diversification in the images. In order to avoid the adverse effect of class imbalances in original dataset, some classes were augmented with artificial images generated using rotation enhancement and brightness enhancement methods.

Using the basic structural framework of state-of-the-art ‘Inception-v3’network, three network architectures were modeled on the maize dataset. We applied baseline learning in these architectures where all the computational layers were trained with our maize dataset. The experimental results state that *Inception-v3_GAP* achieved highest accuracy of 95.99% in separate test dataset. The *Inception-v3_*GAP model was efficient in learning the relevant features from the disease symptoms and in predicting correct class levels in the unseen data. This study proposes that deep learning techniques can provide quite promising results in identifying the disease symptoms of crops. Additionally, this experiment also suggests that in-field images of diseases symptoms of crops with varied background effects can be efficiently modeled by the deep learning techniques without applying any traditional image pre-processing techniques.

Furthermore, to showcase the effectiveness of our proposed approach, we conducted a detailed comparative analysis of a few pre-trained state-of-the-art networks. We used transfer learning approach for the pre-trained models. Comparative results show that *Inception-v3_*GAP model involves higher computational cost in terms of training time and number of parameters than pre-trained models. However, besides high computational cost, *Inception-v3_*GAP model performed quite better at correctly classifying the disease symptoms based on learned features from the data under study. It can be said that baseline training of deep CNN models is also capable of learning low level as well high levels features from the input images and further providing remarkable classification results on the dataset under study.

Moreover, we have validated the model’s disease classification results with the domain experts of maize crop. We are in process of conducting validation of the models in the farmer’s field. In future, the proposed model will be integrated with a mobile application for providing a real-time disease identification tool. This will facilitate a means of automated diagnosis of the disease symptoms to maize growers without any engagement of domain experts or extension workers. Therefore, timely management of diseases and reduction in overall production loss in maize crop will be ensured.

## Supplementary Information

Supplementary Information.

## Data Availability

The datasets used and analysed during the current study are available from the corresponding author on reasonable request.
