# Supplementary material for: Deep learning-based approach for identification of diseases of maize crop
Source: Sci Rep. 2022 Apr 15;12:6334. doi: 10.1038/s41598-022-10140-z (PMC9012772; doi:10.1038/s41598-022-10140-z)
Supplement: Supplementary file 1 — Supplementary Information. [file 41598_2022_10140_MOESM1_ESM.docx]

Supplementary file:

| Table S1: Details of data collection from different maize growing zones | | | | |
| --- | --- | --- | --- | --- |
| **Maize Growing Zones** | **Hotspot Locations** | **Category** | **Year and season** | **Image collection timeline (In days post inoculation (DPI))** |
| Northern Hill Zone  (NHZ) | Almora (Uttarakhand) | Turcicum Leaf Blight | 2019-20;  Kharif | 25-35 and  45-60 |
| North West Plain Zone (NWPZ) | Delhi | Healthy | 2019-20; Rabi and Kharif | 15-25; 30-35 and  45-55 (in DAS) |
|  |  | Maydis Leaf Blight | 2019-20;  (Kharif) | 25-35 and  45-60 |
|  |  | Banded Leaf and Sheath Blight | 2019;  Kharif | 25-35 |
|  | Karnal  (Haryana) | Maydis Leaf Blight | 2020;  Kharif | 25-35 and  45-60 |
|  |  | Banded Leaf and Sheath Blight | 2020;  Kharif | 25-35 and  45-60 |
|  | Ludhiana (Punjab) | Maydis Leaf Blight | 2019-20;  Kharif | 25-35 and  45-60 |
|  |  | Banded Leaf and Sheath Blight | 2019-20;  Kharif | 25-35 and  45-60 |
| North East Plain Zone (NEPZ) | Dholi (Bihar) | Maydis Leaf Blight | 2020  Kharif | 25-35 and  45-60 |
|  |  | Turcicum Leaf Blight | 2019-20;  Rabi | 25-35 and  45-60 |
|  | Kalyani  (West Bengal) | Maydis Leaf Blight | 2019-20;  Rabi | 25-35 |

| **Table S2: Overall accuracies of the models in different data configurations** | | | |
| --- | --- | --- | --- |
| **Data Configuration** | **Inception-v3_flatten-fc** | **Inception-v3_GAP** | **Inception-v3_GAP_fc** |
| 50-35 | 90.84 | 92.12 | 91.80 |
| 55-30 | 90.56 | 93.46 | 92.90 |
| 60-25 | 92.53 | 93.60 | 93.30 |
| 65-20 | 93.39 | 94.61 | 94.28 |
| 70-15 | 95.42 | 95.71 | 95.38 |

Figure S1: Images of diseases of Maize crop A) Healthy B) Maydis Leaf Blight C) Turcicum Leaf Blight and D) Banded Leaf and Sheath Blight


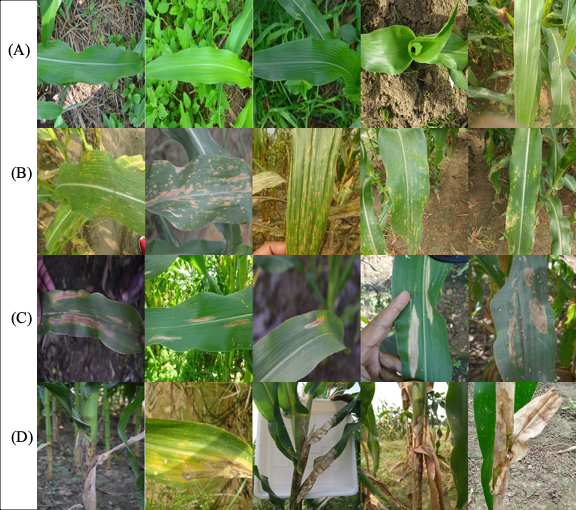


| **Table S3: Comparative performance analysis of the models** | | | | |
| --- | --- | --- | --- | --- |
| **Model** | **Testing Accuracy** | **Average Precision** | **Average**  **Recall** | **Average f1 Score** |
| Inception-v3-GAP | 95.71 | 0.9566 | 0.9568 | 0.9566 |
| VGG 16 | 89.7 | 0.8961 | 0.8966 | 0.8956 |
| VGG 19 | 91.18 | 0.9112 | 0.9109 | 0.9109 |
| Inception-v3 | 72.8 | 0.7341 | 0.7256 | 0.7250 |
| Resnet 50-v2 | 83.74 | 0.8368 | 0.8370 | 0.8359 |
| Resnet 101-v2 | 83.79 | 0.8420 | 0.8380 | 0.8388 |
| Resnet 152-v2 | 85.22 | 0.8420 | 0.8380 | 0.8388 |
| InceptionResnet-v2 | 56.05 | 0.5643 | 0.5553 | 0.5364 |
|  |  |  |  |  |
